# Granulomatous Mastitis Occurring during Pregnancy: A Case Report

**DOI:** 10.3390/medicina59081418

**Published:** 2023-08-03

**Authors:** Ryusei Yoshino, Nana Yoshida, Akane Ito, Nanami Ujiie, Masaki Nakatsubo, Manami Hayashi, Masahiro Kitada

**Affiliations:** 1 Department of Thoracic Surgery and Breast Surgery, Asahikawa Medical University Hospital, 2-1-1-1 Midorigaoka Higashi, Asahikawa-shi 078-8510, Japan; n-takahashi@asahikawa-med.ac.jp (N.Y.); itoakane@asahikawa-med.ac.jp (A.I.); n-ujiie@asahikawa-med.ac.jp (N.U.); tsubo528@asahikawa-med.ac.jp (M.N.); k1111@asahikawa-med.ac.jp (M.K.); 2 Department of Diagnostic Pathology, Asahikawa Medical University Hospital, 2-1-1-1 Midorigaoka Higashi, Asahikawa-shi 078-8510, Japan; kyokui120089@asahikawa-med.ac.jp

**Keywords:** granulomatous mastitis, pregnancy, *Corynebacterium kroppenstedtii*

## Abstract

*Background and Objectives*: Granulomatous mastitis is a benign disease with a clinical presentation similar to that of breast cancer, and is most commonly observed in women of childbearing age. Although it has been suggested that autoimmune diseases are involved in its pathogenesis, no specific treatments have been established. The occurrence of this disease during pregnancy has rarely been reported. We presented the case of a 37-year-old woman who complained of left breast induration at 24 weeks’ gestation. *Materials and Methods*: She was pregnant and manifested a dichorionic, diamniotic placenta. At 24 weeks of gestation, the patient experienced a sensation of hardness in her left breast. Mastitis was suspected, and she was treated with cephem antibiotics. Simultaneously, she was diagnosed with erythema nodosum in the extremities. As her symptoms did not improve, an incisional drainage was performed. Bacterial cultures were obtained at 31 weeks of gestation, and Corynebacterium kroppenstedtii was detected. *Results*: An elective cesarean section was performed at 37 weeks of gestation, and the baby was delivered safely. After delivery, a needle biopsy was performed, and the patient was diagnosed with granulomatous mastitis. She was completely cured with prednisolone after weaning. In this case, the patient’s condition was maintained through incision and drainage, as well as antibiotic, anti-inflammatory, and analgesic drugs during pregnancy. This approach was chosen, taking into consideration the potential side effects of steroids. *Conclusions*: This case suggests that incisional drainage and antibiotic therapy, as well as steroids and surgery, may be considered in the treatment of granulomatous mastitis occurring during pregnancy. This may also be true for management during delivery. After delivery, breastfeeding and steroidal therapy proved to be effective in treating the condition.

## 1. Introduction

Granulomatous mastitis is a benign disease with a clinical presentation similar to that of breast cancer, which often occurs in women of childbearing age, and within two years of childbirth. Although the factors that cause this disease remain unknown, it has been suggested that autoimmune diseases, hyperprolactinemia, oral contraceptives, and pregnancy are involved in its pathogenesis. The clinical manifestations include breast induration and skin inflammation, which often require differentiation from breast cancer. In addition, erythema nodosum has been reported to be associated with the symptoms in many instances [1,2,3].

Puncture aspiration cytology and needle biopsy are useful methods for diagnosis. According to a recent report, granulomatous mastitis is associated with a *Corynebacterium kroppenstedtii* infection. Treatment includes surgical resection, conservative antibiotic therapy, and steroids if symptoms do not improve. However, there are currently no established treatments for this disease, granulomatous mastitis frequently recurs, and the treatment time may be more than one year, making it difficult to treat [2].

There are limited reports on the treatment of granulomatous mastitis during pregnancy. Herein, we describe a case of granulomatous mastitis that occurred during pregnancy and was difficult to treat. In this case, the patient’s symptoms improved with continued incision, drainage, and antibiotics until a cesarean section was performed. This was followed by breastfeeding and steroid treatment after delivery. Most cases of granulomatous mastitis during pregnancy have been reported to improve with increasing delivery time. Therefore, we believe that this case is worth reporting, with a discussion of the previous literature.

This study reports on the course of treatment of granulomatous mastitis during pregnancy, which has rarely been reported in the past. This is also a report of a case that was difficult to manage until the patient was in labor, but was successfully cured. Many women are hesitant to use steroids during pregnancy, and we believe that this case report will be of help in cases of granulomatous mastitis occurring during pregnancy that are difficult to manage. This study may accumulate future cases for the treatment of granulomatous mastitis during pregnancy and provide direction for medical and surgical management.

## 2. Case Presentation

The patient was a 37-year-old woman with a history of four pregnancies, including two vaginal deliveries and one spontaneous miscarriage. Her medical history was unremarkable, except for a diagnosis of polycystic ovary syndrome in 2020. She was undergoing infertility treatment, which involved the use of letrozole hormone and timed therapies. After successfully falling pregnant, the patient underwent uterine fundoplication due to the presence of an empty chorionic sac. Fertility treatment continued, resulting in pregnancy in 2021. No abnormalities were found during the initial antenatal checkups at 10 weeks and 6 days, and 20 weeks and 6 days of gestation. It was discovered that the patient was carrying bichorionic and amniotic twins, with two fetal sacs observed in the uterus.

At exactly 24 weeks of gestation, the patient experienced a sensation of hardness in her left breast. After consulting her midwife, she was instructed to avoid nipple stimulation or to employ cooling measures. At 24 weeks and 6 days of gestation, there were no significant changes in her symptoms. Blood samples were taken, revealing a white blood cell count of 14,360/μL (with neutrophils at 88.2%) and a CRP level of 14.75 mg/dL, indicating an elevated inflammatory response. Therefore, despite mastitis being uncommon in patients that are not breastfeeding, we suspected mastitis and initiated treatment with cephem antibiotics and anti-inflammatory analgesics.

**Figure 1:**
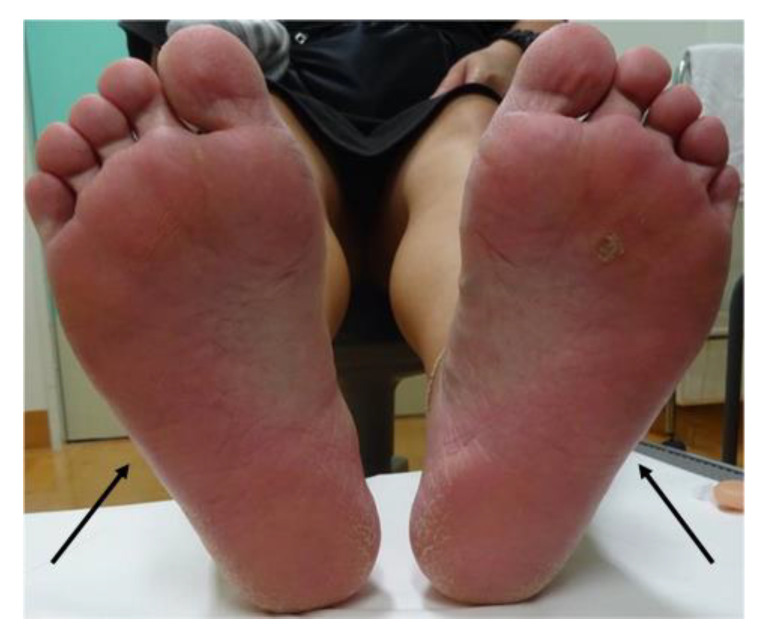
Physical examination. Erythema on bilateral plantar surfaces (arrow).

**Figure 2:**
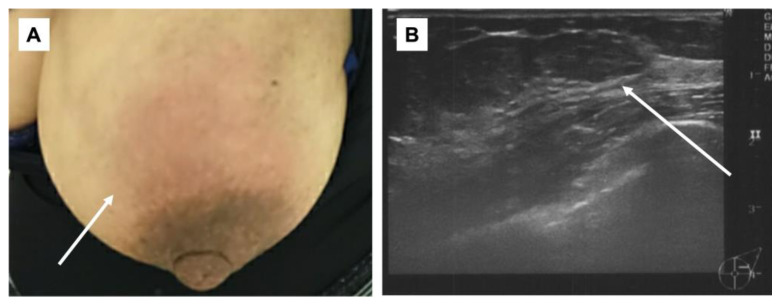
Physical examination/mammary echography. (**A**): Redness was observed in the left breast (arrow); (**B**): internal hypo-absorption area with some dots of hyper-absorption area was observed. The border was clear, and inflammatory disease including abscess was suspected (arrow).

On the second day of the 25th week of pregnancy, the patient developed a tenderness accompanied by erythema on both soles of her feet. Seeking further assistance, she consulted a dermatologist (Figure 1). Initially, the dermatologist diagnosed her with, and treated her for, plantar fasciitis and further prescribed an ointment for treatment. On the same day, the patient was referred to our department for investigation regarding the possible presence of breast cancer owing to a lack of improvement in her symptoms of mastitis. Her height was 155.0 cm and she weighed 59.8 kg. During the physical examination, erythema, pain, and induration were observed in the left breast. Echographic findings suggested pyogenic mastitis (Figure 2A,B). An incision was made to drain the lesion, and a Penrose drain was inserted during the follow-up observation. Cephem antibiotics and anti-inflammatory analgesics were then administered.

At 26 weeks and 2 days of gestation, the patient continued to receive antibiotics with no significant change in symptoms. At 26 weeks and 6 days of gestation, a well-defined painful erythema appeared on her extremities, and a dermatologist diagnosed her with erythema nodosum.

At 28 weeks and 0 days of gestation, she developed a fever, and the symptoms in her left breast had not improved. Therefore, a second incision was made for a Penrose drain insertion. A glucose challenge test was performed as a screening measure during the patient’s pregnancy, and the results raised suspicions of gestational diabetes mellitus. However, due to the presence of an elevated inflammatory response, an oral glucose tolerance test was not performed.

At 29 weeks and 2 days of gestation, the patient’s symptoms improved slightly, but the tenderness persisted. Due to the lack of improvement, a third incisional drainage was performed, and the drained specimen was submitted for bacterial culturing. However, a needle biopsy was not performed because of the suspicion of gestational diabetes mellitus. Aggressive use of steroids was not recommended, and the treatment plan remained largely unchanged.

At 31 weeks and 0 days of gestation, the affected area was well managed, and the approach of incisional drainage and oral antibiotic therapy was continued. The bacterial culture results showed *C. kroppenstedtii*, and the patient was switched from cephem antibiotics to erythromycin (macrolide) based on sensitivity.

At 32 weeks and 0 days of pregnancy, the antibiotic regimen was terminated because the patient was scheduled to undergo a cesarean section, and an elective cesarean section was performed at 37 weeks and 1 day of pregnancy. Following the operation, the patient was treated with gentamicin for four days, clindamycin and vancomycin for five days, and intravenous antibiotic therapy.

**Figure 3:**
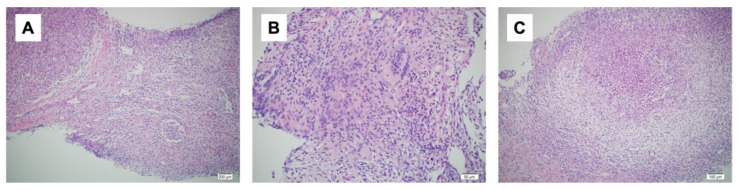
Histopathological findings. (**A**): Non-dysbutyroid epithelial granuloma (hematoxylin-eosin [HE] staining ×10); (**B**): area consisting of a cluster of multinucleated giant cells (hematoxylin-eosin [HE] staining ×20); (**C**): area of an abscess (hematoxylin-eosin [HE] staining ×10).

A day after the elective cesarean section, a bacterial culture and needle biopsy of the left mammary gland were performed. The bacterial culture results again revealed the presence of *C*. *kroppenstedtii.* The needle biopsy revealed granulomatous mastitis (Figure 3A–C). Based on these examination results, the patient was diagnosed with granulomatous mastitis. One month after surgery, the patient’s symptoms flared up again, and she was started on prednisolone 20 mg/day after weaning. Two weeks after starting prednisolone, the dose was reduced to 10 mg/day and the wound healed with iodine ointment. Prednisolone was gradually decreased, the treatment was terminated four weeks after the initial treatment had begun, and the patient was examined during follow up checkups. However, one week later, her symptoms re-emerged and prednisolone was started again at 30 mg/day. The symptoms improved, prednisolone tapered off, and the patient recovered completely after two months. One and a half years after the surgery, no recurrence was observed.

## 3. Discussion

Granulomatous mastitis is a rare disease that typically manifests as a hard and tender mass. It tends to occur within a few years after childbirth. Its occurrence during pregnancy is rarely documented. The most common differential diagnoses include breast cancer, infectious mastitis, vasculitis, and postpartum ductal dilation. There have been reports of granulomatous mastitis occurring in the adnexal breast tissue, and in some cases, it has been difficult to distinguish from breast cancer [1,2,3,4]. The pathogenesis of granulomatous mastitis remains unknown; however, it has been suggested that IgG4-related diseases, autoimmune diseases such as rheumatoid arthritis and hyperprolactinemia, as well as oral contraceptives, and pregnancy may be involved. The involvement of autoimmune diseases has been highlighted because there have been many reports of autoimmune diseases coexisting with granulomatous mastitis and showing a positive response to steroid treatment [5].

There have also been many reports of coexisting erythema nodosum in the lower extremities. The histopathological findings of both granulomatous mastitis and erythema nodosum revealed a composition of multinucleated giant cells, with chronic inflammation and neutrophil infiltration. In addition, the levels of the inflammatory cytokines interleukin (IL)-8, (IL)-22, and (IL)-23 were found to be elevated in patients with granulomatous mastitis. This finding also supports the association between granulomatous mastitis and autoimmune diseases, as patients with autoimmune diseases also have elevated levels of these cytokines [6].

An association between infection of the granulomatous mammary gland and *Corynebacterium* spp. has also been reported [7]. The prevalence of *Corynebacterium* spp. in granulomatous mastitis is high, with *C. kroppenstedtii* being the most frequently identified. In a recent study to identify organisms, amplification of the 16S rRNA and rpoB gene regions was conducted through a polymerase chain reaction. Subsequently, the amplified gene regions were sequenced using matrix-assisted laser desorption/ionization-time of flight mass spectrometry (MALDI-TOF MS), a technique that utilizes laser ionization [2]. Currently, MALDI-TOF MS is routinely used for analysis because of its low cost, high accuracy, and high speed.

In this case, in addition to systemic symptoms such as erythema nodosum and arthritis associated with mastitis-like symptoms, bacterial culture test results were useful in differentiating mastitis from breast cancer. The patient was initially suspected to have mastitis during pregnancy. However, *C*. *kroppenstedtii* was identified through bacterial culture tests, and a needle biopsy led to the diagnosis of granulomatous mastitis. Strictly speaking, granulomatous mastitis is indeed a noninfectious disease, and the role of C. *kroppenstedtii* in this disease remains a matter of debate. However, clinical experience shows that there are many occasions when granulomatous mastitis is difficult to treat, and the search for and identification of *C. kroppenstedtii* by culture testing may help to improve diagnostic certainty. It will also reveal antibiotic susceptibility. It is important to consider the possibility of this disease from the beginning, given the unique identification methods required to reveal the *Corynebacterium* species.

There is no standardized treatment for granulomatous mastitis. In most cases, the basic treatment options are careful observation, antibiotics, steroids, incisional drainage, and a partial mastectomy [2,8]. Although there is limited information available regarding the use of steroids during pregnancy, exposure to steroids during early pregnancy may increase the risk of a fetal cleft lip and palate, preterm delivery, and low birth weight. It is also well known that steroid use increases the risk of Cushing’s syndrome, weight gain, hyperglycemia, and infection [5]. Therefore, it is difficult to treat pregnant and lactating women. An important consideration in the treatment of lactating patients is that oral steroids decrease milk secretion. The other side effects of steroids include insomnia, depression, and agitation. Lactating patients are often physically and emotionally affected, and pain management, including the use of non-steroidal anti-inflammatory drugs and cooling with ice is often important in lactation treatment without the use of steroids. Repeated antibiotic administration is ineffective and may cause unintended side effects. In addition, psychosocial support for the patient’s burden and breastfeeding support are important because of the physical and emotional factors involved [9,10].

In this case, the treatment of granulomatous mastitis during pregnancy, and the cesarean section with incisional drainage and antibiotics, was less invasive and more effective than steroids or surgery. The use of steroids was avoided during pregnancy on account of the consequent side effects, and the patient was followed up with antibiotics and incisional drainage. However, no lessening of the symptoms was observed before delivery.

The infection was well controlled, and after the cesarean section, the patient was treated with gentamicin for four days and clindamycin and vancomycin for five days. During the perioperative period, the patient’s granulomatous mastitis persisted without exacerbation. However, after giving birth, her symptoms resurfaced and were alleviated with a combination of breastfeeding abstinence and oral steroidal therapy. The rarity of a case demonstrating inadequate control of mastitis until delivery makes it worth reporting because it includes the use of antibiotics and breastfeeding abstinence after delivery, and the initiation of steroids. It also serves as a valuable reference for the use of steroids during pregnancy, delivery, and after delivery for patients with this disease who respond poorly to antibiotic treatment.

## 4. Conclusions

In conclusion, systemic symptoms such as erythema nodosum and arthritis, as well as the results of bacterial culture tests, are useful in identifying granulomatous mastitis. Incisional drainage and antibiotic therapy, rather than steroids and surgery, are useful for treating granulomatous mastitis during pregnancy. The condition can also be effectively managed during delivery. Abstinence from breastfeeding and steroidal therapy after delivery have also proven to be effective in managing this disease. The use of steroids during pregnancy should, however, be treated with caution, taking their side effects into consideration.

## Data Availability

Not applicable.
